# ACE Inhibitor and Angiotensin Receptor-II Antagonist Prescribing and Hospital Admissions with Acute Kidney Injury: A Longitudinal Ecological Study

**DOI:** 10.1371/journal.pone.0078465

**Published:** 2013-11-06

**Authors:** Laurie A. Tomlinson, Gary A. Abel, Afzal N. Chaudhry, Charles R. Tomson, Ian B. Wilkinson, Martin O. Roland, Rupert A. Payne

**Affiliations:** 1 Cambridge Clinical Trials Unit, University of Cambridge, Cambridge, United Kingdom; 2 Cambridge Centre for Health Services Research, Institute of Public Health, Cambridge, United Kingdom; 3 Department of Nephrology, Cambridge University Hospitals National Health Service Foundation Trust, Cambridge, United Kingdom; 4 The Richard Bright Renal Unit, North Bristol National Health Service Trust, Bristol, United Kingdom; Charité University Medicine Berlin, Germany

## Abstract

**Background:**

ACE Inhibitors (ACE-I) and Angiotensin-Receptor Antagonists (ARAs) are commonly prescribed but can cause acute kidney injury (AKI) during intercurrent illness. Rates of hospitalization with AKI are increasing. We aimed to determine whether hospital AKI admission rates are associated with increased ACE-I/ARA prescribing.

**Methods and Findings:**

English NHS prescribing data for ACE-I/ARA prescriptions were matched at the level of the general practice to numbers of hospital admissions with a primary diagnosis of AKI. Numbers of prescriptions were weighted for the demographic characteristics of general practices by expressing prescribing as rates where the denominator is Age, Sex, and Temporary Resident Originated Prescribing Units (ASTRO-PUs). We performed a mixed-effect Poisson regression to model the number of admissions for AKI occurring in each practice for each of 4 years from 1/4/2007.

From 2007/8-2010/11, crude AKI admission rates increased from 0.38 to 0.57 per 1000 patients (51.6% increase), and national annual ACE-I/ARA prescribing rates increased by 0.032 from 0.202 to 0.234 (15.8% increase). There was strong evidence (p<0.001) that increases in practice-level prescribing of ACE-I/ARA over the study period were associated with an increase in AKI admission rates. The increase in prescribing seen in a typical practice corresponded to an increase in admissions of approximately 5.1% (rate ratio = 1.051 for a 0.03 per ASTRO-PU increase in annual prescribing rate, 95%CI 1.047-1.055). Using the regression model we predict that 1,636 (95%CI 1,540-1,780) AKI admissions would have been avoided if prescribing rates were at the 2007/8 level, equivalent to 14.8% of the total increase in AKI admissions.

**Conclusion:**

In this ecological analysis, up to 15% of the increase in AKI admissions in England over a 4-year time period is potentially attributable to increased prescribing of ACE-I and ARAs. However, these findings are limited by the lack of patient level data such as indication for prescribing and patient characteristics.

## Introduction

Acute kidney injury (AKI) is a common problem implicated in a substantial proportion of hospital admissions and the incidence is increasing [1]–[3]. It is associated with a marked increase in mortality [1] and also leads to prolonged hospital stay, increased secondary care costs [4] and possibly accelerated decline in long-term kidney function [5].

AKI has many and often multifactorial aetiologies [6]. However, an important cause is the use of ACE inhibitor and Angiotensin-II Receptor Antagonists (ARA) drugs which are associated with AKI in a range of settings, particularly during acute hypovolaemic illness [7]–[13]. The increased risk of AKI among patients taking these medications has been recognised by the UK National Institute for Health and Clinical Excellence (NICE) and the international organisation Kidney Disease: Improving Global Outcomes (KDIGO), both of which recommend that patients with chronic kidney disease (CKD) should stop taking them if they become acutely unwell [14], [15].

There are many evidence based indications for use of ACE inhibitors and ARAs and national guidelines recommend treatment with them for a number of chronic conditions including hypertension, chronic kidney disease with proteinuria, and heart failure with left ventricular dysfunction. The result is that these medicines are the second most commonly prescribed in English primary care, accounting for 6% of all prescriptions [16]. Due to increasing prevalence of chronic comorbidities in older people they are commonly used in the elderly: in Belgium, 7.3% of the population were treated with long-term ACE inhibitors or ARAs and this rose to 36% for people aged 80 years or more [17].

However, despite their frequent use, it is not known to what extent increasing use of these medications has contributed to the increasing incidence of AKI on a population level. This is in part because observational studies on this topic are confounded by indication. The conditions for which ACE inhibitors and ARAs are indicated are themselves associated with increased risk of AKI. Therefore increasing incidence of AKI may reflect increasing prevalence of comorbidities, independently of medications used. We hypothesised that if these medications were playing a causal role, changes in prescribing would be associated with changes in hospital admission with AKI within general practices. We therefore conducted a longitudinal ecological analysis using routinely-collected national hospital administrative data to determine whether hospital admission rates with AKI in England are associated with increased prescribing of ACE inhibitor and ARA therapy.

## Methods

### Data sources

All data used in this study relates to the period 1st April 2007 to 31st March 2011. We used prescribing data from the English National Health Service (NHS) Prescription Services' Prescribing Database (ePACT) [18]. This provides data for each English general practice for the total number of prescriptions that were prescribed and subsequently dispensed, although information about the quantity of medication provided is not captured.

We obtained the numbers of ACE inhibitor (British National Formulary sub-section 2.5.5.1) [19] and ARA prescriptions (British National Formulary sub-section 2.5.5.2) from all general practices in England during the study period. The number of prescriptions for ACE inhibitors and ARAs issued by a general practice will be related to the age and sex demographic of the practice population. Therefore we controlled for differences in general practice populations by expressing prescribing as rates where the denominator is Age, Sex, and Temporary Resident Originated Prescribing Units (ASTRO-PUs) [20]. Because prescribing is generally higher in women and older people, ASTRO-PUs provide a nationally accepted way of weighting prescribing for the age and sex characteristics of the population of a general practice, and thus facilitating the comparison of prescribing between practices. The numbers of ASTRO-PUs for each general practice are updated regularly and a revision to the values of was carried out in April 2008. Therefore, for consistency we used the pre-2008 weightings, devised in 2001, throughout the entire study period. In this study, on average, each person is represented by 4.3 ASTRO-PUs.

We obtained the number of hospital admissions with AKI using data from Hospital Episode Statistics (HES) [21]. HES contains administrative details including diagnostic information on the vast majority of admissions to hospitals in England, coded using the tenth edition of the International Statistical Classification of Diseases and Related Health Problems (ICD-10) [22]. Within HES, each admission is comprised of one or more episodes, with each episode reflecting care by a responsible clinician in a particular hospital ward or department. It is common practice to group episodes occurring close to each other as relating to a continuous period of care (an admission) even if there are small gaps between episodes. We therefore treated episodes occurring within three days of one another as a single admission even if the episodes were recorded under separate admissions (as may happen if patients were transferred between hospitals). The robustness of this approach is examined in a sensitivity analysis (see below).

Each episode records a primary diagnosis (the main condition treated or investigated during this episode) and up to 19 additional secondary diagnoses. For the main analysis defining an AKI admission we required code N17 (acute renal failure) to be present as the primary diagnosis for any episode within seven days of the date of admission. Since ICD-10 was introduced, the term AKI has largely replaced acute renal failure in clinical use but this has not yet been amended for coding purposes. We have previously examined the positive predictive value of code N17 in HES data for the KDIGO definition of AKI and found that for both 2005 and 2010 it was accurate for 95% of cases [23].

### Statistical analysis

We matched the NHS prescribing data to numbers of hospital admissions for AKI at general practice level, aggregated to four one-year periods starting on 1st April 2007. A mixed effect Poisson regression was performed to model the number of admissions for AKI occurring in each practice in each year. Within the model we included prescribing rates (continuous) and year (categorical) as fixed effects variables, together with a random intercept term for general practice. The purpose of the random intercept is to cast the effect of prescribing rates as a within practice, rather than between practice, comparison. In other words the resulting rate ratios can be considered to represent the changes in admission rate when the other variables change for a single practice. The number of person-years at risk was based on the practice population sizes obtained from annual figures recorded as part of the English primary care pay-for-performance scheme, the Quality and Outcomes Framework [24]. A mixed model such as this allows the use of data from general practices where observations are not present for every year (primarily due to general practices closing, opening, and merging during the time period under investigation). To minimize the influence of unusually high prescribing practices on our findings, we excluded general practices with prescribing rates greater than 0.5 ACE inhibitor and ARA prescription per ASTRO-PU.

The relationship between hospital admissions and prescribing was quantified using the change in admissions for an average annual increase in prescribing rate. We also used the model to estimate the number of admissions that would have been avoided in 2010/11 had the prescribing rate for each practice been the same as it had been in 2007/8. In the case of general practices which did not exist in 2007/8, the mean 2007/8 prescribing rate was used in this calculation. Confidence intervals on the number of admissions avoided were calculated using a bootstrap with 100 samples clustered by general practice.

### Sensitivity analyses

To ensure the robustness of our findings, we performed a number of sensitivity analyses.

Over the time course of this study there have been substantial attempts to raise recognition of AKI in hospital patients, as well as altered use of coding to define hospital remuneration. Therefore it is possible that an individual with AKI has a greater probability of this condition being diagnosed and recorded towards the end of the study period, and this may explain to some extent a change in incidence of AKI defined by HES coding. We examined whether our results could be explained by improvements in the thoroughness of clinical coding over time by adjusting for the number of secondary diagnoses recorded (the “coding depth”). Similarly, we examined whether including admissions defined by ICD-10 code N19 (unspecified kidney failure) affected our findings. We examined the effect of restricting the maximum length of included episodes to less than two months, based on the premise that patients may develop AKI during a prolonged admission, despite there being a different primary clinical reason for the admission. Finally, we looked at the effect of altering the overlap of three days that we used to identify a single continuous admission. Further details are given in table S2.

## Results

**Figure 1:**
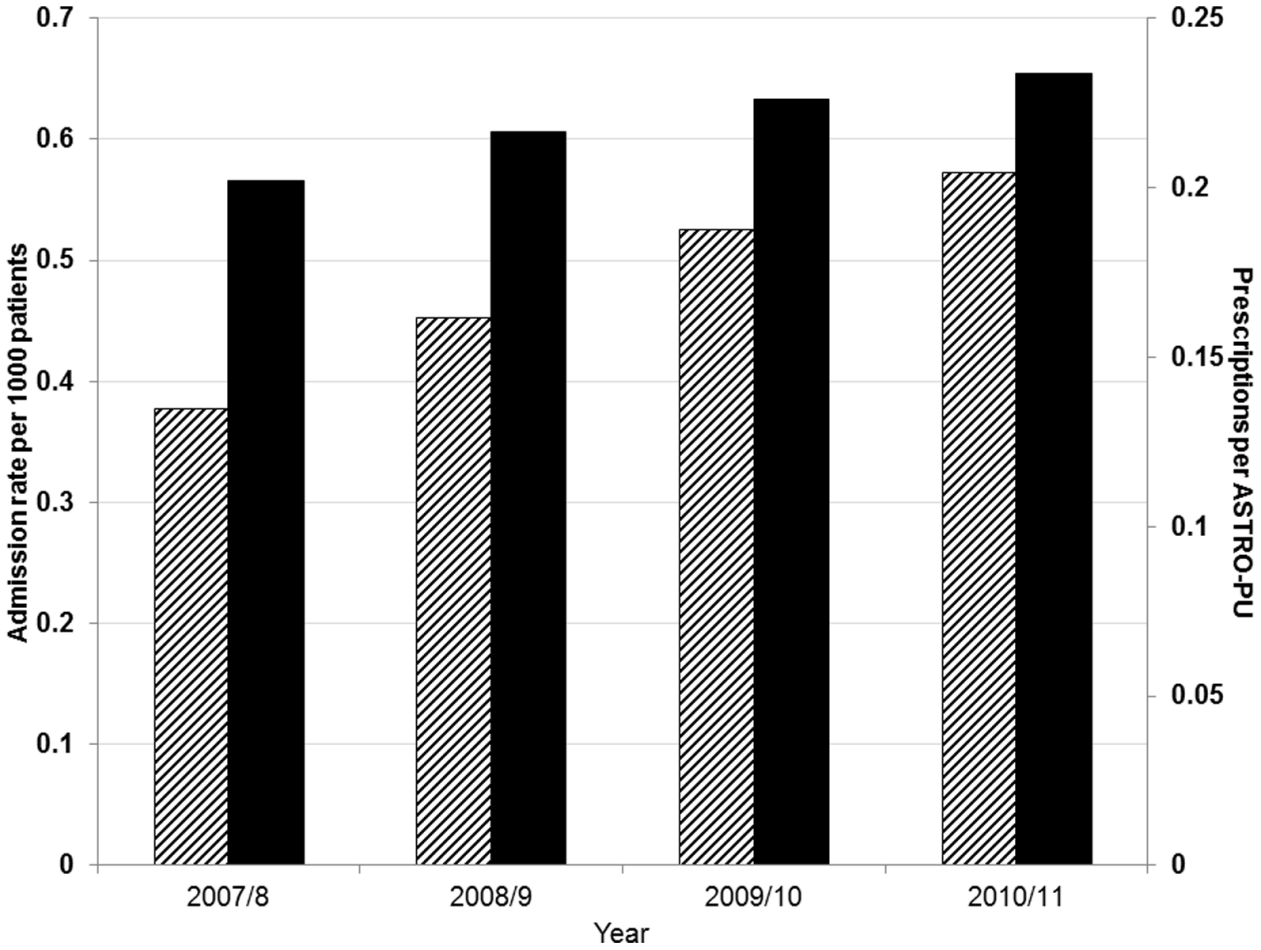
National admission rates of acute kidney injury and prescriptions of ACE Inhibitors and Angiotensin Receptor Antagonists in England between 2007 and 2011. Striped (left) bars represent hospital admission rates; Black (right) bars represent ACE inhibitor and Angiotensin Receptor Blocker prescription rates.

The nationwide changes over time in ACE inhibitor and ARA prescribing and AKI admissions are shown in Table 1 and Figure 1. National annual prescribing rates increased by 0.032 from 0.202 to 0.234 prescriptions per ASTRO-PU over the four years studied (16% increase). Hospital admission rates coded to have AKI in the primary coding position also increased from 0.38 to 0.57 per 1000 patients over the same period, an increase of 52%.

**Table 1:** National figures for number of general practices, total prescribing and total hospital admissions.

	2007/8	2008/9	2009/10	2010/11
Number of general practices	8039	8027	8024	7959
Population (Millions)	53.25	53.68	54.30	54.45
Number of prescriptions (Millions)	46.31	50.19	53.13	55.50
Number of ASTRO-PUs (Millions)	229.15	231.95	235.13	237.52
Prescribing rate (per ASTRO-PU)	0.20	0.22	0.23	0.23
Number of admissions	20118	24314	28555	31180
Admission rate (per 1000 people)	0.38	0.45	0.53	0.57

ASTRO-PU - age, sex and temporary resident adjusted prescribing unit

The number of general practices included in our analysis (i.e. those with both admission and prescribing data, and an ACE inhibitor and ARA prescribing rate less than 0.5 prescriptions per ASTRO-PU) fell from 8039 in 2007/8 to 7959 in 2010/11, although the total population increased from 53.3 to 54.5 million. In any one year, a maximum of 33 general practices (0.4%) were excluded due to the ACE inhibitor and ARA prescribing rate being greater than 0.5 prescriptions per ASTRO-PU.

There were substantial variations in both ACE inhibitor and ARA prescribing rates, and AKI admission rates between general practices (Table S1). For example in 2007/8 the median practice level ACE inhibitor and ARA prescribing rate was 0.20 prescriptions per ASTRO-PU with an interquartile range of 0.15 to 0.25. For AKI admissions the median value was 0.33 admissions per 1000 patients with an interquartile range of 0.13 to 0.54. Similarly the increase in prescribing rate varied considerably between general practices. The median increase was 0.030 prescriptions per ASTRO-PU over the four years studied with an interquartile range of 0.015 to 0.048. The corresponding median increase in number of prescriptions was 882 (IQR 411 to 1616). However, 8.9% of general practices had lower prescribing rates in 2010/11 than in 2007/8.

Results from the Poisson regression model are detailed in Table 2. This shows clear evidence of an increase in AKI admission rates over time (p<0.001), with the average admissions rate 44% higher in 2010/11 than in 2007/8. There is additional strong evidence (p<0.001) that increased ACE inhibitor and ARA general practice prescribing rates were independently associated with increased AKI admission rates, even after the adjustment for the underlying trend over time.

**Table 2:** Association between ACE inhibitor and ARA prescribing and hospital admission with AKI: results from Poisson regression.

Exposure		Rate ratio† (95% confidence interval)
	
ACE inhibitor and ARA prescribing*		1.051 (1.047, 1.055)
Year	2007/8	Ref
	2008/9	1.172 (1.150, 1.194)
	2009/10	1.341 (1.317, 1.366)
	2010/11	1.442 (1.416, 1.469)

* Rate ratio is expressed as change in AKI admission rate for the median general practice increase in prescribing over the study period (0.030 units per ASTRO-PU). ASTRO-PU - age, sex and temporary resident adjusted prescribing unit.

† P<0.001 for all variables.

To quantify this it is helpful to consider the effect associated with the median general practice increase in prescribing over the study period, i.e. 0.030 prescriptions per ASTRO-PU. For such a within practice increase there is on average a 5.1% increase in the hospital admission rate for AKI (rate ratio 1.051, 95% CI 1.047 to 1.055) after adjustment for national increases over time. Using the regression model we predict that 1,636 (95% CI 1,540-1,780) AKI admissions would have been avoided if prescribing rates were at the 2007/8 level. This is equivalent to 14.8% of the total increase in AKI admissions (11,062).

The results of the sensitivity analyses are summarised in table S2. Whilst there are some differences in the effect of year which may reflect changes in coding practice over time, the estimates of the effect of prescribing in all models is very similar to that of the primary analysis indicating that the main finding is robust to our operational definition of an AKI admission.

## Discussion

Over the four-year period of this study, ACE inhibitor and ARA prescribing increased by approximately 16%, and hospital admissions with AKI by just over a half. Our analyses provide strong evidence that, at the level of the general practice, the increase in prescribing is associated with the increase in hospitalisation, and indeed may account for almost 15% of the total increase in AKI admissions.

These findings are consistent with other studies which have demonstrated an increasing incidence of AKI and evidence that AKI can result from treatment with ACE inhibitors and ARAs, usually in the presence of an intercurrent illness. However, it is the first study to quantify the extent to which the changing incidence of AKI may be due to these medications. Studies to examine the association between treatment with ACE inhibitors and ARAs and AKI are difficult since both the drugs and the reason for prescribing them are risk factors for AKI. Patients prescribed and not prescribed the drugs differ in a range of characteristics which are not easily overcome by matching. Previous studies on this topic have tried to overcome this problem through studying interactions between medications [7], [25] or by attempting to control for the indications for prescribing using methods such as propensity scoring or multivariable logistic regression [10], [11], [13]. This is the first ecological study to examine this topic and as such has important strengths including the use of a large, real-world dataset. Because the large majority of England’s population use the state-funded NHS, our study will have captured nearly all relevant prescribing and acute hospital admissions. Our longitudinal study design, incorporating a random effect for practice, also allows us to examine within-practice changes. This overcomes some of the usual problems of ecological analyses, including allowing us to adjust for underlying upward trends in AKI coding. These results add support to the need for carefully designed studies using individual level patient data to examine this issue in more depth.

However, there are also limitations to the analysis. The findings of ecological studies may not reflect individual-level associations and several other factors could explain or contribute to our findings. It is likely that some of the observed increase in hospital admissions with AKI is explained by a higher proportion of cases of AKI being correctly coded due to greater clinical recognition of cases, change in hospital remuneration policies or both. However, this is unlikely to fully explain the associations we have observed since it would not be expected that better hospital coding is associated with changes in prescribing at individual practices. In addition, the findings of the sensitivity analyses examining coding depth provide very similar findings to the main analysis. Our use of hospital administrative coding for AKI is not an ideal measure of incident cases of AKI. Studies of the accuracy of coding for AKI compared to biochemical definitions show that coding has a low sensitivity [26] and can by definition only capture more serious, hospitalized cases. However, we have previously shown that this code, where present, is accurate.

Secondly, the results may also be explained in part by ageing of the population which leads to both increased prescribing of ACE inhibitors and ARAs, and increased risk of AKI. However, our prescribing data is adjusted for the changing age profile of individual general practices. Alternately, an increase in prevalence of comorbidities such as heart failure and chronic kidney disease, again associated with AKI and with prescribing of ACE inhibitors and ARAs, might also be expected to explain some of the association. However, it is likely that any increase in prevalence will be small over the four-year period in question. No accurate data exists to estimate prevalence of these conditions at the level of general practices so we were not able to adjust for them.

Finally, increased use of ACE inhibitors and ARAs may be a marker for increased use of other drugs causally associated with AKI such as diuretics and non-steroidal anti-inflammatory drugs (NSAIDs). Lack of individual patient level data meant that it was not possible to adjust for use of these medications. Nonetheless, there are strong reasons to consider a causal explanation for our findings and if an association between prescribing and admissions at an individual level exists, due to multiple sources of measurement error our practice-level analysis is likely to have underestimated the strength of this association. While the effect size is small this does equate to a substantial number of potentially preventable cases of AKI nationally. It is important to note that the model does not estimate the proportion of AKI admissions attributable to ACE inhibitors and ARAs but the *increase* attributable to the increase in prescribing over this time period. This study covers a time period when use of ACE inhibitors and ARAs were well established, and the increase in prescribing modest. Larger increases in prescribing in the past, associated with the issuing of relevant clinical guidance, may have resulted in a considerably higher proportion of AKI admissions being attributable to these treatments.

The relationship between prescribing of ACE inhibitors and ARAs and AKI is important. There is a substantial body of evidence for the benefits of these drugs for a range of common chronic conditions. In the UK, NICE guidelines advise treatment with them for hypertension, heart failure, ischaemic heart disease and proteinuric chronic kidney disease, including diabetes with minimal proteinuria. We face an ageing population with an increasing burden of multimorbidity, so prescribing of these agents is likely to increase further [27]. Quantification of the benefits of these drugs and subsequent prescribing guidance are determined from randomised clinical trials conducted in select populations with close monitoring. In general clinical use, medications may be used in populations different from that in which trials were conducted and with less frequent patient safety checks [28]. Therefore, adverse effects may be more common in routine clinical practice than in trials and the risk-benefit ratio may be wrongly estimated. In light of this, it is important to investigate this topic more fully in order to improve our understanding of the factors associated with AKI in association with these medications, in order to better risk stratify patients receiving them and to develop evidence-based interventions to prevent this serious complication.

In addition, the evidence regarding drug-associated AKI is predominantly related to high-income countries. However, treatment with ACE inhibitors is recommended by the World Health Organization to prevent onset and delay progression of CKD in low-resource settings [29]. The risk of AKI in low income countries where infectious illness and volume depletion are common is substantial and in the absence of treatment facilities outcomes may be poor [30]. It is therefore vital to ensure that as use of these drugs spreads to other regions, the risk: benefit ratio is reevaluated.

## Conclusion

In England, increased prescribing of ACE inhibitors and ARAs may explain 15% of increased hospital admissions with AKI between 2007 and 2011. Better understanding of individual level risk factors for AKI associated with ACE inhibitors and ARAs are needed to reduce the potential harms associated with these important and commonly prescribed medications. This ecological analysis demonstrates that the national increases in prescribing may be a powerful driver of increased AKI incidence, and throws uncertainty on the balance of benefits and risks associated with use of these drugs.

## Supporting Information

Table S1 **Variation in general practice demographics, practice-level prescribing and hospital admissions across practices.**
(DOCX)

Table S2 **Rate ratios and 95% confidence intervals for AKI admissions for the main and sensitivity analyses.**
(DOCX)

## References

[1] Wang HE, Muntner P, Chertow GM, Warnock DG (2012) Acute Kidney Injury and Mortality in Hospitalized Patients. Am J Nephrol 35: 349–355. 22473149 10.1159/000337487 PMC3362180

[2] Abraham KA, Thompson EB, Bodger K, Pearson M (2012) Inequalities in outcomes of acute kidney injury in England. QJM. 10.1093/qjmed/hcs037 22408153

[3] Hsu CY, McCulloch CE, Fan D, Ordonez JD, Chertow GM, et al (2007) Community-based incidence of acute renal failure. Kidney Int 72: 208–212. 17507907 10.1038/sj.ki.5002297 PMC2673495

[4] Chertow GM, Burdick E, Honour M, Bonventre JV, Bates DW (2005) Acute kidney injury, mortality, length of stay, and costs in hospitalized patients. J Am Soc Nephrol 16: 3365–3370. 16177006 10.1681/ASN.2004090740

[5] Rifkin DE, Coca SG, Kalantar-Zadeh K (2012) Does AKI truly lead to CKD? J Am Soc Nephrol 23: 979–984. 22460531 10.1681/ASN.2011121185 PMC3358766

[6] Bellomo R, Kellum JA, Ronco C (2012) Acute kidney injury. Lancet 380: 756–766. 22617274 10.1016/S0140-6736(11)61454-2

[7] Lapi F, Azoulay L, Yin H, Nessim SJ, Suissa S (2013) Concurrent use of diuretics, angiotensin converting enzyme inhibitors, and angiotensin receptor blockers with non-steroidal anti-inflammatory drugs and risk of acute kidney injury: nested case-control study. BMJ 346: e8525. 23299844 10.1136/bmj.e8525 PMC3541472

[8] Fournier JP, Sommet A, Durrieu G, Poutrain JC, Lapeyre-Mestre M, et al.. (2012) Drug interactions between antihypertensive drugs and non-steroidal anti-inflammatory agents: a descriptive study using the French Pharmacovigilance database. Fundam Clin Pharmacol. 10.1111/fcp.12014 23190210

[9] Loboz KK, Shenfield GM (2005) Drug combinations and impaired renal function – the 'triple whammy'. Br J Clin Pharmacol 59: 239–243. 15676048 10.1111/j.1365-2125.2004.02188.x PMC1884747

[10] Plataki M, Kashani K, Cabello-Garza J, Maldonado F, Kashyap R, et al (2011) Predictors of acute kidney injury in septic shock patients: an observational cohort study. Clin J Am Soc Nephrol 6: 1744–1751. 21734090 10.2215/CJN.05480610

[11] Arora P, Rajagopalam S, Ranjan R, Kolli H, Singh M, et al (2008) Preoperative use of angiotensin-converting enzyme inhibitors/angiotensin receptor blockers is associated with increased risk for acute kidney injury after cardiovascular surgery. Clin J Am Soc Nephrol 3: 1266–1273. 18667735 10.2215/CJN.05271107 PMC2518804

[12] Adhiyaman V, Asghar M, Oke A, White AD, Shah IU (2001) Nephrotoxicity in the elderly due to co-prescription of angiotensin converting enzyme inhibitors and nonsteroidal anti-inflammatory drugs. J R Soc Med 94: 512–514. 11581344 10.1177/014107680109401005 PMC1282204

[13] Rim MY, Ro H, Kang WC, Kim AJ, Park H, et al (2012) The effect of Renin-Angiotensin-aldosterone system blockade on contrast-induced acute kidney injury: a propensity-matched study. Am J Kidney Dis 60: 576–582. 22658321 10.1053/j.ajkd.2012.04.017

[14] National Institute for Health and Clinical Excellence. Chronic kidney disease quality standard (2011) Available: http://publications.nice.org.uk/chronic-kidney-disease-quality-standard-qs5/quality-statement-7-acute-illness.Accessed 2013 February 25.

[15] Wheeler DC, Becker GJ (2013) Summary of KDIGO guideline. What do we really know about management of blood pressure in patients with chronic kidney disease? Kidney Int 83: 377–383. 23325075 10.1038/ki.2012.425

[16] National Health Service. The Information Centre. Prescription Cost Analysis, England (2011) Available: http://www.ic.nhs.uk/catalogue/PUB05807/pres-cost-anal-eng-2011-tab.zip Accessed 25 February 2013.

[17] Smets HL, De Haes JF, De Swaef A, Jorens PG, Verpooten GA (2008) Exposure of the elderly to potential nephrotoxic drug combinations in Belgium. Pharmacoepidemiol Drug Saf 17: 1014–1019. 18763247 10.1002/pds.1641

[18] National Health Service Prescription Services. Available: http://www.nhsbsa.nhs.uk/3230.aspx

[19] Joint Formulary Committee. British National Formulary (online) London: BMJ Group and Pharmaceutical Press http://www.medicinescomplete.com Accessed 2012 May 25.

[20] Roberts SJ, Harris CM (1993) Age, sex, and temporary resident originated prescribing units (ASTRO-PUs): new weightings for analysing prescribing of general practices in England. BMJ 307: 485–488. 8305014 10.1136/bmj.307.6902.485 PMC1678776

[21] National Health Service. The Information Centre. Hospital Episode Statistics. Available: http://www.hesonline.nhs.uk

[22] World Health Organisation. International Classification of Diseases 10th Edition (1990) Available: http://www.who.int/classifications/icd/en/ Accessed 2012 May 25.

[23] Tomlinson LA, Riding AM, Payne RA, Abel GA, Tomson CR, et al (2013) The accuracy of diagnostic coding for acute kidney injury in England - a single centre study. BMC Nephrol 14: 58. 23496869 10.1186/1471-2369-14-58 PMC3599863

[24] National Health Service. The Information Centre. Quality and Outcomes Framework. Online GP practice results database. Available http://www.qof.ic.nhs.uk/ Accessed 2011 October 26.

[25] Huerta C, Castellsague J, Varas-Lorenzo C, Garcia Rodriguez LA (2005) Nonsteroidal anti-inflammatory drugs and risk of ARF in the general population. Am J Kidney Dis 45: 531–539. 15754275 10.1053/j.ajkd.2004.12.005

[26] Vlasschaert ME, Bejaimal SA, Hackam DG, Quinn R, Cuerden MS, et al (2010) Validity of administrative database coding for kidney disease: a systematic review. Am J Kidney Dis 57: 29–43. 10.1053/j.ajkd.2010.08.031 21184918

[27] Barnett K, Mercer SW, Norbury M, Watt G, Wyke S, et al (2012) Epidemiology of multimorbidity and implications for health care, research, and medical education: a cross-sectional study. Lancet 380: 37–43. 22579043 10.1016/S0140-6736(12)60240-2

[28] Brody H, Light DW (2011) The inverse benefit law: how drug marketing undermines patient safety and public health. Am J Public Health 101: 399–404. 21233426 10.2105/AJPH.2010.199844 PMC3036704

[29] World Health Organisation. Package of Essential Noncommunicable (PEN) Disease Interventions for Primary Health Care in Low-Resource Settings (2010) Available: http://whqlibdoc.who.int/publications/2010/9789241598996_eng.pdf Accessed 2013 February 25.

[30] Okunola O, Akinsola A, Ayodele O (2012) Kidney diseases in Africa: aetiological considerations, peculiarities and burden. Afr J Med Med Sci 41: 119–133. 23185909

