# Supplementary material for: ACE Inhibitor and Angiotensin Receptor-II Antagonist Prescribing and Hospital Admissions with Acute Kidney Injury: A Longitudinal Ecological Study
Source: PLoS One. 2013 Nov 6;8(11):e78465. doi: 10.1371/journal.pone.0078465 (PMC3819379; doi:10.1371/journal.pone.0078465)
Supplement: Table S1 — Variation in general practice demographics, practice-level prescribing and hospital admissions across practices. (DOCX) [file pone.0078465.s001.docx]

**Table S1: Variation in general practice demographics, practice-level prescribing and hospital admissions across practices**

|  | **2007/8** | **2008/9** | **2009/10** | **2010/11** |
| --- | --- | --- | --- | --- |
| **Number of general practices** | 8039 | 8027 | 8024 | 7959 |
| **General practice population** | 5819  (3423, 9029) | 5890  (3455, 9120) | 5941  (3490, 9216) | 6014  (3552, 9315) |
| **Number of prescriptions** | 4720  (2729, 7703) | 5130  (2966, 8326) | 5455  (3141, 8860) | 5725  (3341, 9311) |
| **Number of ASTRO-PUs** | 24444  (13929, 39343) | 24902  (14022, 39895) | 25394  (14241, 40568) | 25836  (14612, 41112) |
| **Prescribing rate (per ASTRO-PU)** | 0.20  (0.15, 0.25) | 0.21  (0.16, 0.27) | 0.22  (0.17, 0.28) | 0.23  (0.18, 0.29) |
| **Number of admissions** | 2  (1, 4) | 2  (1, 4) | 3  (1, 5) | 3  (1, 6) |
| **Admission rate (per 1000 people)** | 0.33  (0.13, 0.54) | 0.40  (0.20, 0.64) | 0.47  (0.25, 0.74) | 0.52  (0.27, 0.82) |

Data are described as median (IQR). Note that figures differ from those shown in table 1 because these represent the median figures from general practices rather than the nation as a whole. ASTRO-PU - age, sex and temporary resident adjusted prescribing unit
