# Supplementary material for: ACE Inhibitor and Angiotensin Receptor-II Antagonist Prescribing and Hospital Admissions with Acute Kidney Injury: A Longitudinal Ecological Study
Source: PLoS One. 2013 Nov 6;8(11):e78465. doi: 10.1371/journal.pone.0078465 (PMC3819379; doi:10.1371/journal.pone.0078465)
Supplement: Table S2 — Rate ratios and 95% confidence intervals for AKI admissions for the main and sensitivity analyses. (DOCX) [file pone.0078465.s002.docx]

**Table S2. Rate ratios and 95% confidence intervals for AKI admissions for the main and sensitivity analyses**

| **Exposure** | | **Primary**  **Analysis** | **Sensitivity Analyses†** | | | | | | |
| --- | --- | --- | --- | --- | --- | --- | --- | --- | --- |
|  |  |  | **1** | **2** | **3** | **4** | **5** | **6** | **7** |
|  | |  |  |  |  |  |  |  |  |
| **ACE inhibitor and ARA prescribing*** | | 1.051  (1.047, 1.055) | 1.043  (1.040, 1.046) | 1.045  (1.042, 1.048) | 1.054  (1.049, 1.060) | 1.052  (1.048, 1.056) | 1.045  (1.041, 1.049) | 1.043  (1.040, 1.047) | 1.052  (1.048, 1.056) |
|  |  |  |  |  |  |  |  |  |  |
| **Year** | **2007/8** | Ref | Ref | Ref | Ref | Ref | Ref | Ref | Ref |
|  | **2008/9** | 1.172  (1.150, 1.194) | 1.213  (1.202, 1.225) | 1.118  (1.109, 1.127) | 1.252  (1.219, 1.286) | 1.179  (1.157, 1.201) | 1.282  (1.266, 1.297) | 1.214  (1.202, 1.226) | 1.179  (1.157, 1.201) |
|  | **2009/10** | 1.341  (1.317, 1.366) | 1.426  (1.413, 1.440) | 1.209  (1.200, 1.218) | 1.622  (1.582, 1.664) | 1.348  (1.323, 1.373) | 1.654  (1.634, 1.673) | 1.429  (1.416, 1.443) | 1.348  (1.323, 1.373) |
|  | **2010/11** | 1.442  (1.416, 1.469) | 1.663  (1.647, 1.678) | 1.324  (1.314, 1.335) | 2.082  (2.031, 2.134) | 1.446  (1.420, 1.473) | 2.183  (2.158, 2.208) | 1.666  (1.650, 1.682) | 1.446  (1.420, 1.473) |

P <0.001 for all variables and analyses

***** Rate ratio is expressed as change in AKI admission rate for the median general practice increase in prescribing over the study period (per 0·030 units per ASTRO-PU). ASTRO-PU - age, sex and temporary resident adjusted prescribing unit.

**† Details of sensitivity analyses are as follows:**

1. N17 as a primary or secondary diagnosis;
2. N17 or N19 as a primary or secondary diagnosis;
3. N17 as a primary diagnosis, with five or fewer secondary diagnoses recorded;
4. N17 as a primary or secondary diagnosis, with five or fewer secondary diagnoses recorded;
5. N17 as a primary or secondary diagnosis in episodes of less than two months duration;
6. N17 as a primary diagnosis in episodes of less than two months duration, but not considering episodes occurring in close succession as a single admission;
7. N17 as a primary diagnosis, but considering any episodes occurring within 30 days of one another to relate to the same admission.
